# Correlates of poor medication adherence in chronic psychotic disorders

**DOI:** 10.1192/bjo.2020.141

**Published:** 2020-12-28

**Authors:** Martha Sajatovic, Jessie Mbwambo, Isaac Lema, Carol Blixen, Michelle E. Aebi, Betsy Wilson, Godwin Njiro, Christopher J. Burant, Kristin A. Cassidy, Jennifer B. Levin, Sylvia Kaaya

**Affiliations:** Department of Psychiatry, Department of Neurology, Case Western Reserve University School of Medicine, University Hospitals Cleveland Medical Center, Ohio, USA; Department of Psychiatry and Mental Health, School of Medicine, Muhimbili University of Health and Allied Sciences, Tanzania; Department of Psychiatry and Mental Health, School of Medicine, Muhimbili University of Health and Allied Sciences, Tanzania; Department of Psychiatry and Neurological and Behavioral Outcomes Center, Case Western Reserve University School of Medicine, Ohio, USA; Department of Psychiatry, Case Western Reserve University School of Medicine and University Hospitals Cleveland Medical Center, Ohio, USA; Department of Psychiatry, Case Western Reserve University School of Medicine and University Hospitals Cleveland Medical Center, Ohio, USA; Department of Psychiatry and Mental Health, School of Medicine, Muhimbili University of Health and Allied Sciences, Tanzania; Frances Payne Bolton School of Nursing, Case Western Reserve University, Ohio, USA; Department of Psychiatry, Case Western Reserve University School of Medicine and University Hospitals Cleveland Medical Center, Ohio, USA; Department of Psychiatry and Neurological and Behavioral Outcomes Center, Case Western Reserve University School of Medicine and University Hospitals Cleveland Medical Center, Ohio, USA; Department of Psychiatry and Mental Health, School of Medicine, Muhimbili University of Health and Allied Sciences, Tanzania

**Keywords:** Antipsychotics, psychosis, schizophrenia, medication, treatment adherence

## Abstract

**Background:**

Chronic psychotic disorders (CPDs) occur worldwide and cause significant burden. Poor medication adherence is pervasive, but has not been well studied in sub-Saharan Africa.

**Aims:**

This cross-sectional survey of 100 poorly adherent Tanzanian patients with CPD characterised clinical features associated with poor adherence.

**Method:**

Descriptive statistics characterised demographic and clinical variables, including barriers to adherence, adherence behaviours and attitudes, and psychiatric symptoms. Measures included the Tablets Routine Questionnaire, Drug Attitudes Inventory, the Brief Psychiatric Rating Scale, the Clinical Global Impressions scale, the Alcohol Use Disorders Identification Test and Alcohol, Smoking and Substance Involvement Screening Test. The relationship between adherence and other clinical variables was evaluated.

**Results:**

Mean age was 35.7 years (s.d. 8.8), 61% were male and 80% had schizophrenia, with a mean age at onset of 22.4 (s.d. 7.6) years. Mean proportion of missed CPD medication was 64%. One in ten had alcohol dependence. Most individuals had multiple adherence barriers. Most clinical variables were not significantly associated with the Tablets Routine Questionnaire; however, in-patients with CPD were more likely to have worse adherence (*P* ≤ 0.01), as were individuals with worse medication attitudes (Drug Attitudes Inventory, *P* < 0.01), higher CPD symptom severity levels (Brief Psychiatric Rating Scale, *P* < 0.001) and higher-risk use of alcohol (Alcohol Use Disorders Identification Test, *P* < 0.001).

**Conclusions:**

Poorly adherent patients had multiple barriers to adherence, including poor attitudes toward medication and treatment, high illness acuity and substance use comorbidity. Treatments need to address adherence barriers, and consider family supports and challenges from an intergenerational perspective.

Chronic psychotic disorders (CPDs), such as schizophrenia and schizoaffective disorder, occur worldwide and cause significant burden, characterised by reduced quality of life, functional impairment and premature mortality owing to suicide and other causes. Although the standard of care for CPD includes antipsychotic drugs, poor medication adherence is pervasive globally and leads to negative outcomes that affect not just individuals with schizophrenia, but also families and communities.^1,2^ There are many barriers to medication-taking generally among people with schizophrenia, including erratic medication-taking, poor insight into illness, cognitive problems, stigma and limited access to care.^1,3–5^ A recent systematic literature review and meta-analysis of the global literature on medication non-adherence in major psychiatric disorder suggests that poor adherence is influenced by a variety of factors, such as patients’ individual behaviours; social or family support; clinical, illness and/or treatment factors, and overall healthcare system-related factors.^6^

In sub-Saharan Africa, poor adherence is seen in approximately half of individuals with CPD; however, clinical correlates of poor adherence have not been well studied.^7–11^ A qualitative study from Tanzania^10^ suggested that both patients and caregivers perceived non-adherence to antipsychotic medication as a leading factor of relapse. A literature review on antipsychotic treatment for people with schizophrenia in sub-Saharan Africa, by Chidarikire et al,^1^ noted that limited access to care, a poor understanding of illness and treatment, limited insight, lower levels of literacy, comorbid substance misuse, the presence of CPD symptoms and medication treatment side-effects can all negatively affect adherence.

To develop and deliver care approaches that may promote improved adherence, it is important to understand the clinical features and adherence barriers for people at greatest risk of poor adherence. This cross-sectional survey of patients with CPD in Tanzania characterised demographic and clinical features that appear to be associated with suboptimal medication adherence.

## Method

### Overview

Data from this analysis is derived from a larger 24-month project, funded by the USA's National Institute of Mental Health, to refine and preliminarily test an intervention intended to promote medication adherence in poorly adherent patients. Methods of the larger project have been described elsewhere.^12^ This analysis assessed clinical features and adherence correlates among 100 poorly adherent patients with CPD who were receiving care in the psychiatry department at a 70-bed national referral hospital located in urban Dar es Salaam, Tanzania. Demographic features, past resource use, substance use comorbidity and psychosocial support were evaluated with respect to their association with self-reported medication treatment adherence.

### Study setting and recruitment

Adults aged ≥18 years, with a clinical diagnosis of schizophrenia or schizoaffective disorder, were recruited from the national psychiatric referral hospital and associated hospital ambulatory clinics. Patients at the hospital are referred from four catchment zones that includes three regional public and private hospitals. The out-patient clinic serves mainly discharged patients, for follow-up. Inclusion criteria were purposely broad to capture a representative sample of poorly adherent patients, and included a clinical diagnosis of schizophrenia or schizoaffective disorder, confirmed by a psychiatrist; self-reported poor medication adherence, defined as missing ≥20% of prescribed antipsychotic medication within the past week or past month;^2^ and being able to participate in assessment procedures and be rated on standardised rating scales. Individuals at risk of harm to themselves or others, and those who could not provide written informed consent to study participation, were excluded. The study was approved by the local institutional review board and the Tanzanian National Institute for Medical Research (ethical approval registry number: DA.282/298/01.C; ClinicalTrials.gov Identifier: NCT04327843).

### Assessments

Evaluations comprised demographic and clinical characteristics relevant to CPD relapse. Demographics included age, gender, marital status, children/number of children, educational status, employment and where the individual resided (with family, alone, *etc*.). Clinical variables included type and duration of CPD, family psychiatric history, personal history of physical or sexual abuse, past hospital admissions and antipsychotic medication treatments.

Adherence with CPD medication in the past week and the past month (represented as proportion or percentage of days with a missed medication dose) was assessed with the Tablets Routine Questionnaire (TRQ).^13,14^ The TRQ ranges from 0% (missed no medications) to 100% (missed all medications). The ten-item Drug Attitudes Inventory (DAI)^15^ assessed attitudes toward CPD medications, with scoring calibrated on a 0 (worse attitudes) to 10 (best attitudes) spectrum. CPD symptoms were assessed with the Brief Psychiatric Rating Scale (BPRS).^16^ Global psychopathology was assessed with the Clinical Global Impressions scale (CGI).^17^ Life and work functional status was assessed with the Social and Occupational Functioning Scale (SOFAS).^18^ Substance use or misuse was assessed with the Alcohol Use Disorders Identification Test (AUDIT)^19^ and the Alcohol, Smoking and Substance Involvement Screening Test (ASSIST).^20^ Although there is overlap between these two substance use instruments, the AUDIT specifically focuses on alcohol use patterns whereas the ASSIST evaluates a range of substances, including alcohol, tobacco, cannabis, cocaine, amphetamines, inhalants, sedatives and hallucinogens.

### Medication adherence barriers

In preparation for a future phase of this research project involving pilot testing of an adherence promotion intervention,^12^ we assessed study participants on four main barriers to medication adherence: inadequate or incorrect understanding of CPD medication and consequences of missing medication, non-adherence related to substance use, limited communication with providers to address management of feared or experienced side-effects, and erratic medication-taking and/or lack of medication routines. The focus on these barriers and assessment process, drawn from iterative pilot work,^5,21–23,40^ assessed an individual's specific attitudinal adherence barriers with selected items from the Rating of Medication Influences (ROMI)^24^ and the Attitudes Toward Mood Stabilizers Questionnaire (AMSQ).^25,26^

### Data analysis

We conducted descriptive statistics to characterise demographic and clinical variables, including the number of barriers to adherence. Because gender differences have been documented in treatment adherence and other variables among patients with CPD,^27^ we compared clinical characteristics of men and women in this sample, using chi-squared and two-tailed *t*-tests, reporting effect sizes. Correlational analyses with Spearman correlations (because of the non-normal distribution of lifetime hospital admissions) were conducted to evaluate the association between TRQ and demographic and clinical variables, as well as the relationship between lifetime number of hospital admissions for psychiatric illness and demographic and clinical variables. A multiple linear regression analysis was performed to assess the effects of in-patient versus out-patient status and DAI, BPRS, SOFAS and AUDIT scores on adherence over the past month, as measured by the TRQ.

## Results

### Sample description

Table 1 shows demographic and clinical variables in this poorly adherent sample. Notably, this was a relatively young sample (mean age 35.70 years, s.d. 8.80), with a slight preponderance of men (61%). The majority (80%) had schizophrenia, with an average age at onset of 22.38 (s.d. 7.64) years. The great majority of individuals lived with family (84%) and just over half (57%) had children. Most were in-patients (83%) at the time of assessment, and had an average of 4.72 (4.30) previous hospital admissions for psychiatric illness. Most (>80%) were prescribed first-generation antipsychotic drugs, with oral haloperidol being the most common medication therapy. Family history of mental illness and substance misuse were common, at rates of 53% and 43%, respectively. Compared with men, women with CPD were less likely to live with family (*P* < 0.01, *w* = 1.185), more likely to have a mood component to their CPD (schizoaffective diagnosis, *P* = 0.03, *w* = 0.706), had fewer lifetime hospital admissions for psychiatric illness (*P* = 0.02, *d* = 0.501) and had a greater likelihood of past sexual abuse (*P* = 0.01, *w* = 0.602).

**Table 1:** Demographic and clinical characteristics of poorly adherent individuals with CPD in Dar es Salaam, Tanzania

Variable	Mean (s.d.) or *n* (%), *N* = 100	Male, *n* = 61	Female, *n* = 39	Statistic (d.f.)^a^
Age, years	35.70 (8.80), range 19–62	36.26 (9.28)	34.82 (8.04)	*t*(98) = 0.80, *P* = 0.43
Marital status				
Single, never married	58 (58.0%)	38 (62.3%)	20 (51.3%)	*χ*^2^(2) = 2.44, *P* = 0.30
Married	28 (28.0%)	17 (27.9%)	11 (28.2%)	
Separated/divorced/widowed	14 (14.0%)	6 (9.9%)	8 (20.5%)	
Children (*n* = 99)				
Have children	57 (57.0%)	30 (49.2%)	27 (69.2%)	*χ*^2^(1) = 3.58, *P* = 0.60
Do not have children	42 (42.0%)	30 (49.2%)	12 (30.8%)	
Number of children (*n* = 99)	1.39 (1.81), range 0–10	1.18 (1.71)	1.72 (1.93)	*t*(97) = −1.44, *P* = 0.15
Education level, years (*n* = 99)	9.42 (3.61), range 2–19	9.00 (3.44), *n* = 60	10.08 (3.83)	*t*(97) = −1.46, *P* = 0.15
Employment				
Full time	23 (23.0%)	10 (16.4%)	13 (33.3%)	*χ*^2^(2) = 5.67, *P* = 0.06
Part time	29 (29.0%)	22 (36.1%)	7 (17.9%)	
Unemployed	48 (48.0%)	29 (47.5%)	19 (48.7%)	
Primary residence				
Lives alone	11 (11.0%)	5 (8.2%)	6 (15.4%)	*χ*^2^(2) = 10.07, *P* < 0.01
Lives with family	84 (84.0%)	56 (91.8%)	28 (71.8%)	
Other	5 (5.0%)	0 (0.0%)	5 (12.8%)	
Diagnosis				
Schizophrenia	80 (80.0%)	53 (86.9%)	27 (69.2%)	*χ*^2^(1) = 4.63, *P* = 0.03
Schizoaffective	20 (20.0%)	8 (13.1%)	12 (30.8%)	
Age at CPD onset	22.38 (7.64), range 1–49	21.82 (7.65)	23.26 (7.63)	*t*(98) = −0.92, *P* = 0.36
Illness duration, years	12.39 (7.99), range 1–36	13.46 (8.30)	10.72 (7.28)	*t*(98) = 1.69, *P* = 0.10
Treatment status at assessment				
In-patient	83 (83.0%)	52 (85.2%)	31 (79.5%)	Fisher's exact test, *P* = 0.59
Out-patient	17 (17.0%)	9 (14.8%)	8 (20.5%)	
CPD medication prescribed				
Chlorpromazine	8 (8.0%)	5 (8.2%)	3 (7.7%)	*χ*^2^(4) = 2.42, *P* = 0.66
Haloperidol	72 (72.0%)	46 (75.4%)	26 (66.7%)	
Olanzapine	11 (11.0%)	6 (9.9%)	5 (12.8%)	
Risperidone	8 (8.0%)	4 (6.6%)	4 (10.3%)	
Trifluoperazine	1 (1.0%)	0 (0.0%)	1 (2.6%)	
Lifetime hospital admissions				
Psychiatric	4.72 (4.30), median 3.00	5.49 (4.99)	3.51 (2.50)	*t*(98) = 2.30, *P* = 0.02
Substance misuse	0.45 (1.35), median 0.00	0.64 (1.64)	0.15 (0.59)	*t*(98) = 1.77, *P* = 0.08
History of abuse				
Physical (*n* = 99)				
Yes	55 (55.0%)	32 (52.5%)	23 (59.0%)	*χ*^2^(1) = 0.31, *P* = 0.58
No	44 (44.0%)	28 (45.9%)	16 (41.0%)	
Sexual				
Yes	27 (27.0%)	11 (18.0%)	16 (41.0%)	*χ*^2^(1) = 6.38, *P* = 0.01
No	73 (73.0%)	50 (82.0%)	23 (59.0%)	
Family history				
Mental illness				
Yes	53 (53.0%)	34 (55.7%)	19 (48.7%)	*χ*^2^(1) = 0.47, *P* = 0.49
No	47 (47.0%)	27 (44.3%)	20 (51.3%)	
Alcohol misuse				
Yes	43 (43.0%)	26 (63.4%)	17 (43.6%)	*χ*^2^(1) = 0.01, *P* = 0.92
No	57 (57.0%)	35 (57.4%)	22 (56.4%)	

CPD, chronic psychiatric disorder.

a. Two-tailed *t*-test or chi-squared comparison between males and females.

Table 2 shows scores on standardised rating scales in the sample. The proportion of missed CPD medication in the past week/month was extensive, with patients missing an average of nearly two-thirds of prescribed medication in the past month. CPD symptom scores were relatively low. Approximately three-quarters of the sample were at low risk for having problems with substance misuse; however, one in ten had alcohol dependence, which was the most common substance of misuse. Substances other than alcohol and tobacco were not widely used. Based on the AMSQ and ROMI assessments, the most common adherence barrier domains were inadequate or incorrect understanding of the CPD and poor communication with providers. Approximately one in three had adherence barriers related to the use of substances.

**Table 2:** Standardised rating scale scores of poorly adherent Tanzanian patients with CPD

Variable	Mean (s.d.) or *n* (%), *N* = 100	Male, *n* = 61	Female, *n* = 39	Statistic	Effect size
TRQ					
Past week	89.87 (21.77), range 14–100	94.38 (15.70), range 29–100	82.82 (27.59), range 14–100	*P* = 0.0209^a^	*d* = 0.515
Past month	64.44 (37.50), range 6–100	73.23 (36.18), range 13–100	50.69 (36.16), range 6–100	*P* = 0.0030^a^	*d* = 0.623
DAI	2.98 (4.56), range −10 to 10	2.69 (4.98), range −10 to 10	3.44 (3.84), range −4 to 10	*P* = 0.4270^a^	–
BPRS	34.64 (8.24), range 19–58	34.95 (7.78), range 19–53	34.15 (8.98), range 20–58	*P* = 0.6393^a^	–
CGI	2.52 (0.97), range 1–5	2.49 (0.96), range 1–5	2.56 (0.99), range 1–5	*P* = 0.7179^a^	–
SOFAS	64.24 (9.24), range 41–90	62.75 (8.46), range 48–85	66.56 (10.03), range 41–90	*P* = 0.0438^a^	*d* = 0.411
AUDIT					
Mean (s.d.), median, range	5.38 (8.03), median 0.00, range 0–31	7.00 (8.32), median 4.00, range 0–29	2.85 (6.91), median 0.00, range 0–31	*P* = 0.0109^a^	*d* = 0.543
Severity/risk categories					
Low risk	72 (72.0%)	37 (60.7%)	35 (89.7%)	*P* = 0.0061^b^	*w* = 0.133
Risky/hazardous level	15 (15.0%)	14 (23.0%)	1 (2.6%)		
High risk/harmful level	4 (4.0%)	3 (4.92%)	1 (2.6%)		
Almost certainly dependent on alcohol^c^	9 (9.0%)	7 (11.48%)	2 (5.1%)		
ASSIST					
Global risk	5.79 (3.67), median 6.00, range 0–12	7.28 (2.97), median 9.00, range 0–12	3.46 (3.48), median 3.00, range 0–12	*P* < 0.0001^a^	*d* = 1.181
Tobacco	7.96 (10.13), median 3.00, range 0–32	11.62 (10.61), median 9.00, range 0–32	2.23 (5.87), median 0.00, range 0–28	*P* < 0.0001^a^	*d* = 1.095
Alcohol	9.76 (10.36), median 3.00, range 0–34	12.61 (10.60) median 9.00, range 0–34	5.31 (8.31), median 3.00, range 0–31	*P* = 0.0004^a^	*d* = 0.766
Cannabis	4.33 (7.90), median 0.00, range 0–31	6.44 (8.86), median 3.00, range 0–21	1.03 (4.53), median 0.00, range 0–28	*P* = 0.0006^a^	*d* = 0.769
Other	0.73 (3.03), median 0.00, range 0–21	1.05 (3.81), median 0.00, range 0–21	0.23 (0.81), median 0.00, range 0–3	*P* = 0.1095^a^	*d* = 0.298
Adherence barriers^d^					
Limited understanding of CPD/CPD treatment	100 (100.0%)	61 (100.0%)	39 (100.0%)	Not applicable	Not applicable
Poor communication with providers	100 (100.0%)	61 (100.0%)	39 (100.0%)	Not applicable	Not applicable
Erratic medication routines	97 (97.0%)	59 (96.7%)	38 (97.4%)	*P* = 1.00^e^	–
Substance misuse as an impediment to adherence	35 (35.0%)	27 (44.3%)	8 (20.5%)	*P* = 0.0185^e^	*w* = 0.986

CPD, chronic psychotic disorder; TRQ, Tablets Routine Questionnaire; DAI, Drug Attitudes Inventory; BPRS, Brief Psychiatric Rating Scale; CGI, Clinical Global Impressions scale; SOFAS, Social and Occupational Functioning Scale; AUDIT, Alcohol Use Disorders Identification Test; ASSIST, Alcohol, Smoking and Substance Involvement Screening Test.

a. Independent samples *t*-test.

b. Fisher's exact test.

c. If AUDIT total score is ≥20 and the sum of questions 4–6 are ≥4, it is likely the person is almost certainly dependent on alcohol.

d. Adherence barriers (range 1–4) identified as a focus of treatment in the customised adherence enhancement (CAE curriculum).

e. Chi-squared.

### Adherence correlates

Table 3 shows correlates of adherence over the past month, as measured by the TRQ. Demographic and most clinical variables were not significantly associated with TRQ levels; however, in-patients with CPD were more likely to have worse adherence (*r* = −0.26, *P* ≤ 0.01), as were individuals with worse drug attitudes (*r* = −0.28, *P* < 0.01), higher CPD symptom severity levels (*r* = 0.36, *P* < 0.001), worse functioning (*r* = −0.21, *P* < 0.05) and higher-risk use of alcohol (AUDIT score, *r* = 0.34, *P* < 0.001).

**Table 3:** Spearman correlations between adherence over the past month (measured by TRQ), demographics and standardised rating scales

Variable	*r*_s_ (*P-*value)
Age	0.01 (0.34)
Married versus not married^a^	−0.01 (0.89)
Children versus do not have children (*n* = 99)^b^	−0.05 (0.60)
Number of children (*n* = 99)^b^	0.01 (0.92)
Educational level (*n* = 99)^b^	0.13 (0.22)
Employed versus unemployed	−0.04 (0.72)
Lives alone versus with others	0.08 (0.46)
Schizophrenia versus schizoaffective	−0.11 (0.26)
Age at onset	0.03 (0.77)
Illness duration	−0.13 (0.20)
In-patient versus out-patient^c^	−0.26 (<0.01)
Hospital admissions for psychiatric illness	−0.18 (0.07)
Substance misuse hospital admissions	0.04 (0.71)
Physical abuse versus none (*n* = 99)^b^	−0.06 (0.58)
Sexual abuse versus none	0.05 (0.63)
Family mental illness versus none	−0.06 (0.57)
Family alcohol misuse versus none	0.01 (0.95)
On haloperidol versus other medication	−0.06 (0.55)
DAI^c^	−0.28 (<0.01)
BPRS^c^	0.36 (<0.001)
CGI-Global	0.04 (0.70)
SOFAS^c^	−0.21 (0.04)
AUDIT^c^	0.34 (<0.001)

TRQ, Tablets Routine Questionnaire; DAI, Drug Attitudes Inventory; BPRS, Brief Psychiatric Rating Scale; CGI, Clinical Global Impressions scale; SOFAS, Social and Occupational Functioning Scale; AUDIT, Alcohol Use Disorders Identification Test.

a. Marital status categories were collapsed such that ‘not married’ included those who were single/never married as well as those who were separated, divorced or widowed.

b. Ninety-nine participants completed the survey.

c. In-patient status is significantly correlated with a higher (worse) TRQ, more negative medication attitudes (lower DAI score) are associated with higher (worse) TRQ, more severe psychiatric symptoms are associated with higher (worse) TRQ, worse (lower) social functioning is associated with higher (worse) TRQ, more substance use problems are associated with higher (worse) TRQ.

Using the five significant predictors at the bivariate level, we tested a multiple linear regression to determine which predictors contributed the most to adherence. In-patient status (standardised *β* = −0.219, unstandardised *β* = −21.872, s.e. 0.088, *P* < 0.05), worse symptom severity (standardised *β* = 0.211, unstandardised *β* = −1.529, s.e. 0.103, *P* < 0.05) and higher-risk use of alcohol (standardised *β* = 0.241, unstandardised *β* = 1.130, s.e. 0.092, *P* < 0.05) were found to be associated with higher TRQ scores for adherence over the past month. DAI and SOFAS scores were not significant. The model explained 26.1% of the variance in adherence.

### Hospital admission correlates

Tables 4 and 5 show correlates of lifetime number of hospital admissions for demographic/clinical variables and standardised ratings, respectively. As seen in Table 4, males (*P* = 0.038), those with an earlier age at onset (*P* = 0.049), longer illness duration (*P* < 0.001) and those with children (*P* = 0.047) had significantly more hospital admissions for psychiatric illness. BPRS, CGI, AUDIT, DAI and TRQ scores were not associated with number of lifetime hospital admissions. Table 5 shows results of the multiple linear regression analysis assessing the effects of in-patient versus out-patient status and DAI, BPRS, SOFAS and AUDIT scores on adherence over the past month, as measured by the TRQ. In the linear regression analysis, there was a significant association between adherence and in-patient status, BPRS and AUDIT scores.

**Table 4:** Spearman correlations between demographic variables and number of lifetime hospital admissions for psychiatric illness (*N* = 100)

Variable	*r*_s_ (*P*-value)
Age	0.19 (0.06)
Male (versus female)	−0.21 (0.038)
Educational level	−0.07 (0.48)
Earlier age at onset (versus later)	−0.20 (0.049)
Longer illness duration (versus shorter)	0.38 (<0.001)
Individuals with children (versus without children)	−0.20 (0.047)
History of physical abuse (versus no abuse)	−0.16 (0.11)
History of sexual abuse (veruss no abuse)	−0.12 (0.24)
Married^a^ (versus not married)	0.06 (0.52)
Family history of mental illness (versus none)	0.04 (0.70)
Family history of alcohol misuse (versus none)	0.08 (0.46)
Employed^a^ (versus not employed)	−0.01 (0.94)
Lives alone^a^ (versus lives with family)	−0.01 (0.93)

Men had significantly more hospital admissions compared with women. Those with children were more likely to have hospital admissions versus those without children. Those with earlier age at onset and longer duration of illness had significantly more hospital admissions for psychiatric illness.

a. Collapsed variable to dichotomous format.

**Table 5:** Associations from multiple linear regression models for past month medication adherence (TRQ)

	Unstandardised *β*	s.e.	Standardised *β*	s.e.	*P*-value
In-patient versus out-patient	−21.872	9.25	−0.219	0.09	0.020
DAI	−1.529	0.77	−0.185	0.10	0.055
BPRS	0.966	0.47	0.211	0.10	0.043
SOFAS	−0.082	0.41	−0.020	0.10	0.842
AUDIT	1.130	0.43	0.241	0.09	0.012

TRQ, Tablets Routine Questionnaire; DAI, Drug Attitudes Inventory; BPRS, Brief Psychiatric Rating Scale; SOFAS, Social and Occupational Functioning Scale; AUDIT, Alcohol Use Disorders Identification Test.

## Discussion

The cross-sectional assessment of poorly adherent patients with CPD characterised demographic and clinical variables associated with medication adherence. It must be noted that the sample was specifically enrolled because they were known to be poorly adherent, and findings may not generalise to the broader population of people with CPD in this setting, including those who are adherent with prescribed medications. For the most part, and aligned with reports from settings outside of sub-Saharan Africa,^6^ a number of demographic and clinical variables were not significantly associated with adherence levels; however, those with the worst adherence were most likely to be in-patients with more severe CPD symptoms, have substance use comorbidity and endorse negative attitudes toward CPD medications. Most individuals appear to have multiple barriers to adherence, including attitudinal characteristic that may be a potential target of future interventions.

Reasons for poor adherence in Tanzania include low health literacy, lack of access to medicines and structural factors (e.g. long distances to clinics to collect medicines, clinic overcrowding and low proportion of staff to patients). Similar to our sample, a review by Chidarikire^1^ noted that the majority of people with schizophrenia were treated with first-generation antipsychotics that may cause motor and neurological side-effects, which are an impediment to adherence.^28–30^ Although this analysis did not quantitatively assess for medication side-effects or medication preferences, recent qualitative work conducted by this team on a subset of patients in this sample found that burdensome side-effects with prescribed antipsychotic drugs included weight gain, weakness/tiredness and loss of libido,^31^ all negatively affecting treatment engagement and adherence. Accommodating patient preferences for specific treatments may be particularly challenging in settings where fewer types of medication options might be practical and available.

The average rate of missed CPD medication in this Tanzanian sample was 64%, with an average of four or five past hospital admissions for psychiatric illness in just over a decade of having CPD. Other reports have documented the close association between poor adherence and poor outcomes, including relapse, leading to hospital admission and treatment-resistant illness.^32,33^ The findings from this sample suggest that most poorly adherent patients experience barriers to adherence in multiple domains, and it seems likely that care approaches will need to target patient attitudes/beliefs about medication, communication with providers to address treatment expectations and discussions regarding side-effects, and managing medication in the context of lifestyle and routines.

The in-patient setting may be an appropriate place to identify individuals at highest risk of poor adherence, and this might be an opportunity to provide education on the role of CPD medication in recovery. This may also be an opportunity to implement long-acting injectable antipsychotic medication for individuals who have a known history of poor adherence or those who miss medication as a result of forgetting. Recent studies have suggested that long-acting injectable antipsychotic medication can significantly reduce relapse rates among individuals with schizophrenia.^34^

Adherence promotion approaches in sub-Saharan Africa and elsewhere also need to consider comorbidity as an impediment to optimal medication adherence. At least one in ten poorly adherent individuals with CPD in our sample had alcohol dependence, and about one in three had clinically relevant problems with use/misuse of substances. Thus, adherence and recovery efforts ideally need to include treatment for substance use disorders as part of a comprehensive package of care.

Our analysis found gender differences in selected clinical variables, including health resource use, with men more likely to be admitted to hospital. Differences in premorbid or early illness progression, also reported in the literature, might explain the fact that men had more lifetime hospital admissions compared with women.^35,36^ It is possible that addressing treatment adherence soon after CPD diagnosis might help with reducing hospital admissions, a particularly important goal in low-resource settings.

An additional factor that needs to be considered is the incorporation of family challenges and support in helping patients to self-manage treatment and adherence. The overwhelming majority (84%) of individuals in this sample lived with family, and more than half had children of their own. Substance misuse and family history of both mental illness and substance misuse were common, and a pragmatic consideration would be to address the issue of mental health from an intergenerational perspective. Although our study did not find any correlation between living with family or living alone and treatment non-adherence, a Tanzanian qualitative study conducted by Sariah et al^10^ suggested that family and peer support, employment and religion were perceived as ‘protective’ elements to minimise patient risk of relapse episodes. Family therapies, including psychoeducation, have been demonstrated as improving outcomes and family burden in CPD,^37–40^ and culturally informed approaches could be helpful as an augmentation to adherence-promotion interventions.

This study has a number of methodological limitations that must be considered with respect to both interpretation and generalisability of findings. We did not survey an adherent group of patients, and findings on poorly adherent patients may not apply to those across the full range of adherence. Given the severe paucity of data on antipsychotic drug adherence in sub-Saharan African populations with CPD, this study was exploratory and not designed or intended to be powered to examine gender differences in CPD or the specific variables that might be associated with worse adherence, such as substance use or personal trauma history. Given this exploratory nature, we did not control for multiple comparisons, and the cross-sectional design does not permit for conclusion of causal inferences. The study methods also do not permit a clear assessment of structural barriers to adherence, which are noted in the review by Chidarikire et al^1^ to include poor access to care as a result of poverty or other factors, lack of education or poor health literacy and problems in patient–clinician communication. These factors need to be more specifically addressed in future investigations.

## Data Availability

Scientists interested in accessing the data should reach out to the lead author (M.S.). Data access is subject to national USA and Ugandan data use agreement terms.

## Supplementary material

For supplementary material accompanying this paper visit http://dx.doi.org/10.1192/bjo.2020.141. click here to view supplementary material
